# Comparison of Oil Content and Fatty Acids Profile of Western Schley, Wichita, and Native Pecan Nuts Cultured in Chihuahua, Mexico

**DOI:** 10.1155/2018/4781345

**Published:** 2018-01-22

**Authors:** L. R. Rivera-Rangel, K. I. Aguilera-Campos, A. García-Triana, J. G. Ayala-Soto, D. Chavez-Flores, L. Hernández-Ochoa

**Affiliations:** Facultad de Ciencias Químicas, Universidad Autónoma de Chihuahua, Campus Universitario #2, Circuito Universitario, 31125 Chihuahua, CHIH, Mexico

## Abstract

Two different extraction processes, Soxhlet and ultrasound, were used to obtain the oil extracts of Western Schley, Wichita, and Native pecan nuts cultured in Chihuahua, Mexico. The aspects evaluated in this study were the extraction yield of the processes and fatty acids' profile of the resulting extracts. Gas chromatography coupled with mass spectrometry (GC-MS) was used to identify and determine the composition percentage of fatty acids present in pecan nuts oils extracted. The results obtained show that higher oil extraction yields were obtained by Soxhlet method with hexane (69.90%) in Wichita varieties. Wichita, Western Schley, and Native pecan nuts from Chihuahua are rich in PUFA (polyunsaturated fatty acids) and MUFA (monounsaturated fatty acids) and have low levels of SFA (saturated fatty acids). The predominant fatty acid present in all pecan nuts oils was linoleic acid followed by oleic acid. Myristic acid, palmitic acid, and linolenic acid were also identified in representative quantities. The results from this study suggest that there are statistically significant differences in the chemical composition of the pecan nuts oils extracted from the varieties cultured in Chihuahua, Mexico, and those cultivated in other regions of the world.

## 1. Introduction

In recent years, a lot of interest has been given to the development of edible oils which are rich in bioactive compounds used in health and nutrition industries. An example of such oils is the one obtained from pecan nuts. Pecans [*Carya illinoinensis* (Wangenh.) K. Koch] are native from North Mexico and south USA and belong to the Juglandaceae family. Chihuahua, Mexico, offers suitable conditions for the cultivation of high-quality pecan nut varieties. Chihuahua is the world leader in production of pecan nut with 80000 tons cultured in 2015; Delicias district produces 53% of Chihuahua's pecan nut. Predominantly three varieties of nuts are cultivated in Delicias district: Western Schley, Wichita, and Native nuts, and their areas sown are 84.9%, 15%, and 0.1%, respectively [1, 2]. The pecan nut oil is considered a healthy oil due to the fact that it is a rich source of unsaturated fatty acids (UFA) containing primarily monounsaturated fatty acids (MUFA), a good quantity of polyunsaturated fatty acids (PUFA), and a low content of saturated fatty acid (SFA) [3, 4]. Pecan kernels total lipid content varies from 65% to 75% of total kernel weight. Lipid content reports of Western Schley and Wichita varieties in Australia are 73.08% and 73.45%, respectively, while native nut located in central Mexico is 73.95% [5, 6]. In pecan nut oil, the content of oleic acid (C18:1n-9) is the highest followed by linoleic acid (C18:2n-6), lower concentrations of palmitic acid (C16:0), stearic acid (C18:0), and alfa-linolenic acid (C18:3n-3) and traces of other eight fatty acids as seen in analysis of Tunisian-grown and Australian-grown pecan nuts oils obtained by Soxhlet method with petroleum ether [6, 7]. Western Schley nut oil fatty acid content is 53.38% of oleic acid, 34.24% of linoleic acid, 6.65% of palmitic acid, 2.57% of stearic acid, and 1.74% of alfa-linolenic acid. Fatty acid profile of Wichita nut oil is 57.28% of oleic acid, 31.50 of linoleic acid, 6.56% of palmitic acid, 2.38% of stearic acid, and 1.73% of alfa-linolenic acid. Western Schley and Wichita nut oil determinations were done with southeast Australian-grown pecan nuts, using Soxhlet with petroleum ether as extraction method [6]. Fatty acid composition of native nut oil extracted from central Mexican-grown pecan nuts by Soxhlet method with hexane is 64.55% of oleic acid, 24.40% of linoleic acid, 5.23% of palmitic acid, 2.71% of stearic acid, and 2.21% of alfa-linolenic acid [5]. The intake of oils rich in unsaturated fatty acids is associated with many health benefits. Recent studies have demonstrated that the consumption of MUFA can lower LDL (low-density lipoprotein) cholesterol, protects against coronary heart disease (CHD), regulates the blood pressure, and may have beneficial effects on coagulation factors, inflammation, and endothelial activation [8, 9]. Likewise, the consumption of PUFA has positive effects in reducing the risk of cardiovascular and inflammatory diseases [10]. In addition, PUFA have antithrombotic and antiatherosclerotic properties [11]; they may prevent the development of diseases like arterial hypertension and insulin resistance [12, 13]. Also, PUFA have shown protective effects against diabetic renal disease [14]. Despite the health benefits that can be provided for pecan nuts and their economic importance, the chemical components of pecan nuts cultured in Delicias district, Chihuahua, have not been characterized yet. The objective of this study was to characterize the fatty acid profile by GC/MS of Western Schley, Wichita, and Native pecan nut varieties cultured in Delicias district, Chihuahua, Mexico.

## 2. Materials and Methods

### 2.1. Pecan Samples

Pecan nuts were mechanically harvested during fall (October-November) 2015 at Delicias district, Chihuahua. Western Schley, Wichita, and Native cultivars were chosen due to their commercial relevance. Approximately 2 kg of nuts per cultivar was dried to 5–7% of humidity and stored at 4°C until the oil extraction process. For analysis, 1 kg of nuts per cultivar was manually cracked and the kernel that included the pellicle was separated from the shell. Kernels were immediately processed.

### 2.2. Chemicals

HPLC grade solvents (hexane, ethanol, and methanol), boron trifluoride (BF_3_), NaOH, and NaCl were purchased from Sigma Chemical Co. (St. Louis, MO).

### 2.3. Oil Extraction

50 g of nuts per variety was minced using a food grinder prior to use. Two extraction methods were considered: ultrasound and Soxhlet.

*Ultrasound-Assisted Extraction.* 50 g of minced nuts per variety was mixed separately with 250 ml of solvent (ethanol or hexane) in a 600 ml beaker. The pecan nut solvent suspension was ultrasonicated for 2 hours using a 50 kHz ultrasonic bath (BRANSON 5800). Suspensions were kept in a water bath at 25°C during sonication and were continuously stirred. Suspensions were filtered with a Buchner funnel and medium filtration rate filter paper (Whatman number 1). The remaining powder was dried at 100°C for 1 hour. Oil was obtained after evaporating solvent with a Rotavapor at 65°C and then measured. The oil was stored at 4°C until analyses.

*Soxhlet Extraction.* 50 g of minced nuts per variety was put separately in a Soxhlet apparatus with 250 ml of solvent (ethanol or hexane). Oil was extracted during 3 hours at 68°C. Oil was obtained by solvent evaporation in a Rotavapor at 65°C and then measured. The oil was stored at 4°C until analyses.

### 2.4. Preparation of Fatty Acid Methyl Esters (FAMEs)

Methyl-esterification of samples used in the analyses was performed by the BF_3_ method after alkaline hydrolysis. 2 mL of 0.5 M NaOH-methanol solution was added to 20 *μ*g of oil sample, and the mixture was heated at 100°C for 7 min. After cooling, 3 mL of 14% BF_3_-MeOH reagent was added, and the tube was sealed and heated at 100°C for 5 min. After cooling, 2 mL of hexane and 7 mL of saturated NaCl solution were added, followed by a thorough shaking. The resulting hexane layer was used as a sample solution for GC [15].

### 2.5. Fatty Acids Profile by GC-MS

The fatty acid profile was evaluated for the different oil samples obtained in the present work by Soxhlet with hexane or ethanol and ultrasound with hexane or ethanol for 3 pecan nut varieties. Analysis of FAME was performed by duplicate on a GC-MS Perkin-Elmer instrument (AutoSystem XL Gas Chromatograph-TurboMass Gold Mass Spectrometer). Separations were achieved using a fused silica PE-1 capillary column (30 m × 0.32 mm ID, 0.25 *μ*m film thickness). Helium was used as the carrier gas at flow rates of 1.0 mL/min and a split ratio of 25 : 1. The injector temperature was 250°C. The oven temperature was programmed at 140°C for a hold of 10 min and programmed to reach 210°C at a rate of 7°C/min and held for 5 min and then programmed to reach 250°C at a rate of 7°C/min. TurboMass software was used to control the operation of GC-MS. MS spectra were obtained at range width *m*/*z* 60–450, interface temperature of 200°C, ion source temperature of 200°C, solvent cut time of 5 min, event time of 0.20, and scan speed of 2500.

### 2.6. Statistical Analysis

The data were analyzed using the analysis of variance (ANOVA). Comparisons of means were done using the Minitab statistical software (version 16). Differences between fatty acid profiles were assessed using Tukey's test. Differences at *p* < 0.05 were considered to be significant. Four determinations were performed on each sample.

## 3. Results and Discussion

The global yield (Xo) values for pecan nut (Wichita, Western Schley, and Native) obtained for the different extraction methods (Soxhlet and ultrasound) are presented in Table 1.

**Figure 1:**
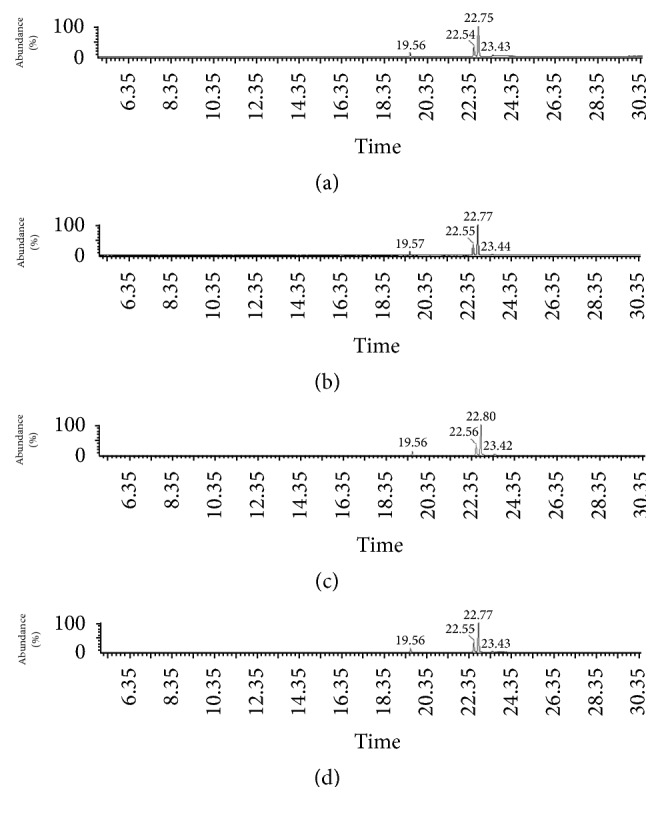
Western Schley, Wichita, and Native pecan nut oil profile determined by GC/MS. Ultrasound method: ethanol (a) and hexane (b); Soxhlet method: ethanol (c) and hexane (d). Retentions time [min]: palmitic acid C16:0 (19.56), oleic acid C18:1 (22.54), linolenic acid C18:3n3 (22.77), and linoleic acid C18:2n-6C18:3n3 (23.43).

**Figure 2:**
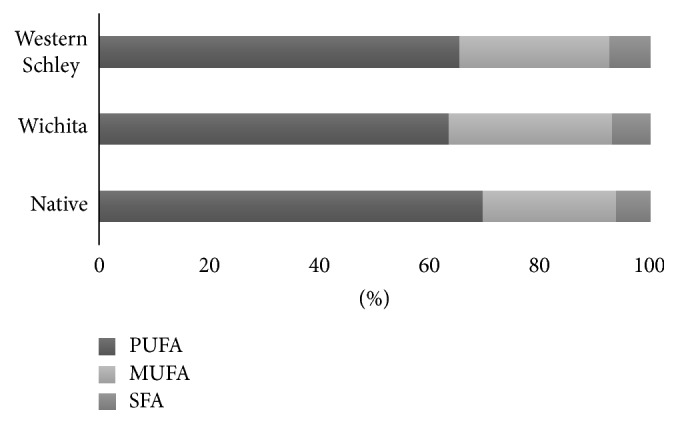
Percentage of monounsaturated fatty acids (MUFA), polyunsaturated fatty acids (PUFA), and saturated fatty acids (SFA) of oil extracted from Western Schley, Wichita, and Native pecan nut varieties.

For Wichita variety, the highest extraction global yield (Xo) was obtained by Soxhlet with hexane (69.900%); for Native pecan, the better extraction yield was achieved using Soxhlet with hexane (67.180%) and for Western Schley an extraction yield of 62.460% was accomplished with Soxhlet method and hexane as solvent (Table 1). The oil yields reported by us are lower compared with those in literature for pecan nut of 72% [16]. Comparing data between pecan varieties, Wichita had higher Xo value followed by Native and Western Schley; these results do not concord with previous reports in which no significant differences were observed in oil content values of distinct varieties of pecan nuts [6]. Soxhlet yields were higher compared to ultrasound method, probably due to solvent recirculation, where the solvent always reaches the sample, renovated and clean [17], and also because of higher-temperature process, enhancing extraction rate [18]. Hexane was more efficient to extract oil than ethanol as it has been reported in seeds oil extractions previously [19]. Gas chromatography coupled with mass spectrometry (GC-MS) was used to identify and determine the composition percentage of fatty acids present in Western Schley, Wichita, and Native pecan nuts oils extracted using ultrasound and Soxhlet methods. Fatty acids detected on oil samples were SFA: lauric acid (C12:0), myristic acid (C14:0), palmitic acid (C16:0), and stearic acid (C18:0); MUFA: palmitoleic acid (C19:1n-9), oleic acid (C18:1), and eicosanoic acid (C20:1n-9); and PUFA: linoleic acid (C18:2n-6) and linolenic acid (C18:3n3). GC-MS chromatograms show four well-defined peaks for palmitic acid (C16:0), oleic acid (C18:1), linoleic acid (C18:2n-6), and linolenic acid (C18:3n3) with retention times of 19.56 min, 22.55 min, 22.77 min, and 23.43 min, respectively. No differences were found in chromatograms among varieties of oils, extraction methods, or solvents (Figure 1). GC-MS revealed that PUFA were predominant over MUFA and SFA; however, SFA has less abundance in all varieties of pecan oils (Figure 2). This result contrasts with most of the previous reports of pecan nut oils [6, 7]; also it has concordance with Ireland-bought and Egyptian-grown pecan nuts oils results and with that fatty acid content of walnut oil [20–22]. Wichita pecan oil SFA, MUFA, and PUFA contents are 6.68% ± 0.95, 29.03% ± 1.42, and 61.96% ± 2.23, respectively; ratios obtained were of PUFA/SFA, 9.43 ± 1.54 : 1, MUFA/SFA, 4.41 ± 0.65 : 1, and UFA/SFA, 13.84 ± 2.18 : 1 (Table 2). Western Schley variety oil contents are SFA, 7.30% ± 1.09, MUFA, 26.80% ± 1.49, and PUFA, 64.55% ± 1.34; ratios for this oil are PUFA/SFA, 9.02 ± 1.61 : 1, MUFA/SFA, 3.71 ± 0.39 : 1, and UFA/SFA, 12.74 ± 1.98 : 1 (Table 2). Native pecan oil has 6.05% ± 0.31 of SFA, 23.92% ± 0.505 of MUFA, and 68.65% ± 1.90 of PUFA; this oil has ratios of PUFA/SFA, 11.36 ± 0.68 : 1, MUFA/SFA, 3.95% ± 0.39 : 1, and UFA/SFA, 15.33 ± 0.89 : 1 (Table 2). Oils obtained from Native and Western Schley varieties showed higher PUFA levels, while Wichita variety oil had the highest MUFA content and no differences were found in SFA percentages. Also, no significant differences were observed in PUFA/SFA, MUFA/SFA, and UFA/SFA ratios between pecan nut oils varieties (Table 2). These results showed that pecan nuts from Chihuahua are rich in MUFA and PUFA and have low content of SFA, this could represent health benefits for humans as LDL cholesterol lowering, protection against coronary heart disease (CHD), regulation of blood pressure, and reduction of the risk of cardiovascular and inflammatory diseases [8–10]. PUFA/SFA, MUFA/SFA, and UFA/SFA ratio values found in this study are higher than others reported in previous studies in which Western Schley, Wichita, and Native pecan nuts were used [7, 23]. Higher PUFA/SFA and UFA/SFA ratios are associated with increasing of the serum HDL-cholesterol and decreasing of plasma insulin levels [24, 25], while MUFA/SFA higher ratio is related to increased physical activity and resting energy expenditure and even to less anger [26]. Fatty acid profiles of pecan nut oils are shown in Table 3. Linoleic acid is the most prevalent fatty acid among varieties of oils followed by oleic acid, palmitic acid, linolenic acid, and myristic acid; traces of lauric, palmitoleic, stearic, and eicosanoic acids were detected in all the oils extracted by Soxhlet and ultrasound methods. Usually oleic acid is the major fatty acid of pecan nut oil followed by linoleic, palmitic, linolenic, stearic, and myristic acids [3]. This was not observed in our study where the percentages vary. Thus, the linoleic acid had the highest quantity followed by oleic, palmitic, linolenic, and myristic acids. Similar data were noted in pecans bought in Irish market and Egyptian-grown Western Schley pecans [20, 21], while reports of Australian-grown Wichita and Western Schley and Tunisian-grown pecan nuts showed that the oleic acid was the major fatty acid present in the oil extracted [6, 7]. These data highlight the role of growing locality on the fatty acid profile. In addition, the pecan composition varies depending on the environment conditions, horticultural practices, cultivar, and season [27]. Traces of vitamin E, vitamin C, and B-carotene also were found in all varieties of oils. These results accord with reports of pecan nut oils analyzed around the world [7, 20, 28].

GC-MS revealed that native pecan oil had the richest content of linoleic acid; it also had the lowest content of oleic acid and this could be related to the biosynthesis of linolenic acid from oleic acid [29]. No significant acid content differences were observed for myristic acid, palmitic acid, and linolenic acid among oil varieties (Table 4).

## 4. Conclusion

The results obtained in this research have defined the benchmark levels for the chemical composition of Wichita, Western Schley, and Native pecan nuts cultured in Chihuahua, Mexico. Wichita pecan variety had the highest oil content, followed by Native pecan and Western Schley pecan variety. The best oil global yield (Xo) in all pecan nut samples was achieved with Soxhlet method and hexane as a solvent. Wichita, Western Schley, and Native pecan nuts from Chihuahua are rich in PUFA and MUFA and have low levels of SFA. PUFA/SFA, MUFA/SFA, and UFA/SFA ratios found in all pecan nuts varieties are higher than those reported in literature and could be related to improvements for human health. The predominant fatty acid present in all pecan nuts oils was linoleic acid followed by oleic acid, which contrasts with most of the data reported. Myristic acid, palmitic acid, and linolenic acid were also identified in representative quantities. Traces of other eight fatty acids, vitamin E, vitamin C, and B-carotene were detected too. Higher and lower contents of linolenic acid and oleic acid, respectively, were found in Native pecan nuts. There do not appear to be other major differences between the three cultivars. Our results differ from those previously published for pecans grown in other parts of the world apparently due to differences in cultivars, environmental conditions, or horticultural practices.

## Figures and Tables

**Table 1:** Global yield (Xo) of extracts from Western Schley, Wichita, and Native pecan nut varieties obtained by two low pressure extraction (LPE) methods and two different solvents.

LPE method	Solvent	Xo (%)^1^		
		Western Schley	Wichita	Native
Soxhlet	Hexane	62.46 ± 0.4^a(a)^	69.90 ± 0.6^a(b)^	67.18 ± 0.4^a(b)^
Soxhlet	Ethanol	41.96 ± 0.5^b^	45.52 ± 0.3^b^	40.78 ± 0.7^b^
Ultrasound	Hexane	17.17 ± 1.1^c^	17.92 ± 0.9^c^	19.19 ± 1.2^c^
Ultrasound	Ethanol	16.04 ± 1.1^c^	14.37 ± 1.1^d^	10.19 ± 0.8^d^

Each value is mean ± standard deviation (SD) of a triplicate extraction; same letters in the same column indicated no significant difference at level of 5% (*p* < 0.05); same letter in the parenthesis in the same row indicated no significant difference at level of 5% (*p* < 0.05). ^1^Dry basis.

**Table 2:** Monounsaturated fatty acids (MUFA), polyunsaturated fatty acids (PUFA), and saturated fatty acids (SFA) percentages and PUFA/SFA, MUFA/SFA, UFA/SFA ratios of pecan nut oil extracted from Western Schley, Wichita, and Native pecan nut varieties.

Cultivar	PUFA	MUFA	SFA	PUFA/SFA	MUFA/SFA	UFA/SFA
Western Schley	64.55 ± 1.34^ab^	26.80 ± 1.49^b^	7.30 ± 1.09^a^	9.02 ± 1.61 : 1^a^	3.71 ± 0.39 : 1^a^	12.74 ± 1.98 : 1^a^
Wichita	61.96 ± 2.23^a^	29.03 ± 1.42^a^	6.68 ± 0.95^a^	9.43 ± 1.54 : 1^a^	4.41 ± 0.65 : 1^a^	13.84 ± 2.18 : 1^a^
Native	68.65 ± 1.90^b^	23.92 ± 0.505^c^	6.05 ± 0.32^a^	11.36 ± 0.68 : 1^a^	3.95 ± 0.27 : 1^a^	15.33 ± 0.89 : 1^a^

Each value is mean ± standard deviation (SD); same letters in the same column indicated no significant difference at level of 5% (*p* < 0.05).

**Table 3:** Fatty acid profile from Western Schley, Wichita, and Native pecan nut oils considering the extraction method and solvent.

Pecan nut variety	Solvent	Method	SFA		MUFA	PUFA	
			C14:0	C16:0	C18:1n-9	C18:2n-6	C18:3n-3
Western Schley	Hexane	Ultrasound	0.066%	5.830%	24.560%	64.709%	1.800%
Western Schley	Hexane	Soxhlet	0.022%	7.710%	27.583%	62.420%	1.930%
Western Schley	Ethanol	Ultrasound	0.025%	7.065%	27.516%	61.618%	1.905%
Western Schley	Ethanol	Soxhlet	0.025%	8.444%	27.540%	62.060%	1.780%
Wichita	Hexane	Ultrasound	0.011%	5.780%	29.057%	62.653%	2.340%
Wichita	Hexane	Soxhlet	0.111%	7.830%	27.650%	59.170%	0.680%
Wichita	Ethanol	Ultrasound	0.023%	6.810%	30.990%	60.549%	1.568%
Wichita	Ethanol	Soxhlet	0.132%	6.020%	28.440%	59.070%	1.800%
Native	Hexane	Ultrasound	0.045%	5.535%	24.300%	66.047%	2.360%
Native	Hexane	Soxhlet	0.030%	6.193%	23.300%	68.270%	2.050%
Native	Ethanol	Ultrasound	0.074%	6.154%	24.360%	64.920%	1.152%
Native	Ethanol	Soxhlet	0.014%	6.166%	23.710%	68.026%	1.780%

Percentages may not add to 100% due to other constituents not listed. Fatty acid detected: C14:0, myristic acid; C16:0, palmitic acid; C18:1n-9, oleic acid; C18:2n-6, linoleic acid; C18:3n-3, linolenic acid. SFA: saturated fatty acids; MUFA: monounsaturated fatty acids; PUFA: polyunsaturated fatty acids.

**Table 4:** Tukey pairwise comparisons with 95% of confidence for fatty acids detected in oils extracted from Western Schley, Wichita, and Native pecan nut varieties.

Fatty acid	Cultivars		
	Western Schley	Wichita	Native
Myristic acid C14:0	0.03450 ± 0.01822^a^	0.06925 ± 0.05294^a^	0.04075 ± 0.02210^a^
Palmitic acid C16:0	7.26225 ± 0.96037^a^	6.61000 ± 0.80084^a^	6.01200 ± 0.27575^a^
Oleic acid C18:1n-9	26.7997 ± 1.29334^a^	29.0343 ± 1.23437^a^	23.9175 ± 0.43774^b^
Linoleic acid C18:2n-6	62.7017 ± 1.19318^b^	60.3605 ± 1.45100^b^	66.8157 ± 1.39323^a^
Linolenic acid C18:3n-3	1.85375 ± 0.06474^a^	1.59700 ± 0.59895^a^	1.83550 ± 0.53802^a^

Same letters in the same line indicated no significant difference at level of 5% (*p* < 0.05).

## Abbreviations

SFA: Saturated fatty acid
PUFA: Polyunsaturated fatty acids
MUFA: Monounsaturated fatty acids
UFA: Unsaturated fatty acids
LDL: Low-density lipoprotein.
